# Investigation of predictors for invasive pulmonary aspergillosis in patients with severe fever with thrombocytopenia syndrome

**DOI:** 10.1038/s41598-023-28851-2

**Published:** 2023-01-27

**Authors:** Li Song, Yingjie Zhao, Gang Wang, Wenlu Zou, Lintao Sai

**Affiliations:** 1 Department of Infectious Diseases, Qilu Hospital of Shandong University, Wenhua Xi Road 107, Jinan, 250012 Shandong China; 2 Department of Rheumatology, Qilu Hospital of Shandong University, Wenhua Xi Road 107, Jinan, 250012 Shandong China

**Keywords:** Fungal infection, Viral infection

## Abstract

Patients with severe fever with thrombocytopenia syndrome (SFTS) had been confirmed to have immune dysfunction and were prone to invasive pulmonary aspergillosis (IPA), which was directly related to the increased mortality. The aim of this study was to investigate the predictors for IPA in SFTS patients, and the results were expected to be helpful for early identification of IPA and initiation of anti-fungal therapy. The study was performed to review laboratory confirmed SFTS patients in two tertiary hospitals in Shandong province (Qilu Hospital of Shandong University and Shandong Public Health Clinical Center) from April 2021 to August 2022. The enrolled patients were further divided into IPA group and non-IPA group. Demographic characteristics, clinical manifestations and laboratory parameters between IPA group and non-IPA group patients were analyzed and compared to identify the independent predictors for IPA by univariate analysis and multivariable logistic regression analysis. Sensitivity and specificity of independent predictors were evaluated by receiver operating characteristic (ROC) curve analysis. In total, 67 SFTS patients were enrolled with an average age of 64.7 (± 8.4) years old. The incidence of IPA was 32.8% (22/67). Mortality of patients in IPA group was 27.3% (6/22), which was significantly higher than that in non-IPA group. Results of univariate analysis showed that uncontrolled diabetes, central nervous system symptoms, platelet < 40 × 10^9^/L, CD4^+^ T cell < 300/μL and CD8^+^ T cell < 400/μL were risk factors for development of IPA. These factors were further analyzed by multivariable logistic regression analysis and the results indicated that uncontrolled diabetes, platelet < 40 × 10^9^/L, CD4^+^ T cell < 300/μL and CD8^+^ T cell < 400/μL could be recognized as independent predictors for IPA in SFTS patients. In conclusion, IPA is a serious complication for SFTS patients and increases mortality. It is necessary to early identify predictors of IPA for improving survival of SFTS patients.

## Introduction

Severe fever with thrombocytopenia syndrome (SFTS) is an emerging infectious disease, which was first reported in China in 2010 and confirmed to be caused by a novel *bunyavirus* (severe fever with thrombocytopenia syndrome virus, SFTSV)^1^. SFTS is characterized by fever, leukopenia and thrombocytopenia. The course of this infectious disease is generally divided into three stages: fever stage, critical stage and recovery stage^2^. Severe complications usually occur in the critical stage, which may lead to multiple organs failure and increase mortality. In the early epidemics, the average annual mortality of SFTS was 8.2% from 2010 to 2013^3^. Another surveillance showed that the average annual mortality of SFTS reduced to a stable level about 5.2% from 2010 to 2019^4^. Given the high mortality, SFTSV has been included in the list of priority target pathogens requiring urgent attention by the World Health Organization (WHO)^5^.

SFTS patients can have multiple systemic complications, in which invasive pulmonary aspergillosis (IPA) is one of the most serious complications. IPA as a common type of invasive aspergillosis usually occurs in immunocompromised patients with neutropenia, transplantation, hematological malignancy or long term use of corticosteroids^6^. Severe SFTS patients had been confirmed to have immune dysfunction including leukopenia and reduction of immune cells^7^. Some studies had reported that SFTS patients were prone to IPA, which was directly related to the increased mortality^8–10^. Therefore, early identification of IPA in SFTS patients is necessary. In order to improve survival of SFTS patients, this study was performed to confirm the predictors for IPA in SFTS patients in their early stage of disease. The results are expected to be helpful for the early identification of IPA and initiation of anti-fungal therapy.

## Methods

### Study design

To confirm the predictors for IPA in SFTS patients, we analyzed demographic feature, clinical manifestations and laboratory parameters of 67 laboratory confirmed SFTS patients from two tertiary hospitals (Qilu Hospital of Shandong University and Shandong Public Health Clinical Center) in Shandong province between April 2021 and August 2022. This study was a purely retrospective study. The clinical course without any additional intervention was reviewed to analyze the risk relationship between related risk factors and the occurrence of IPA. This study was approved by Medical Ethical Committee in Qilu Hospital of Shandong University (2021–120) and written informed consent was acquired from every enrolled patients or their guardians. All methods were performed in accordance with the Declaration of Helsinki and the relevant guidelines and regulations.

### Patients enrollment and grouping

SFTS patients were diagnosed and enrolled according to the following criteria: (i) clinical presentation with acute fever and thrombocytopenia; (ii) serum positive for SFTSV RNA detected by real-time polymerase chain reaction (RT-PCR) assay.

**Figure 1:**
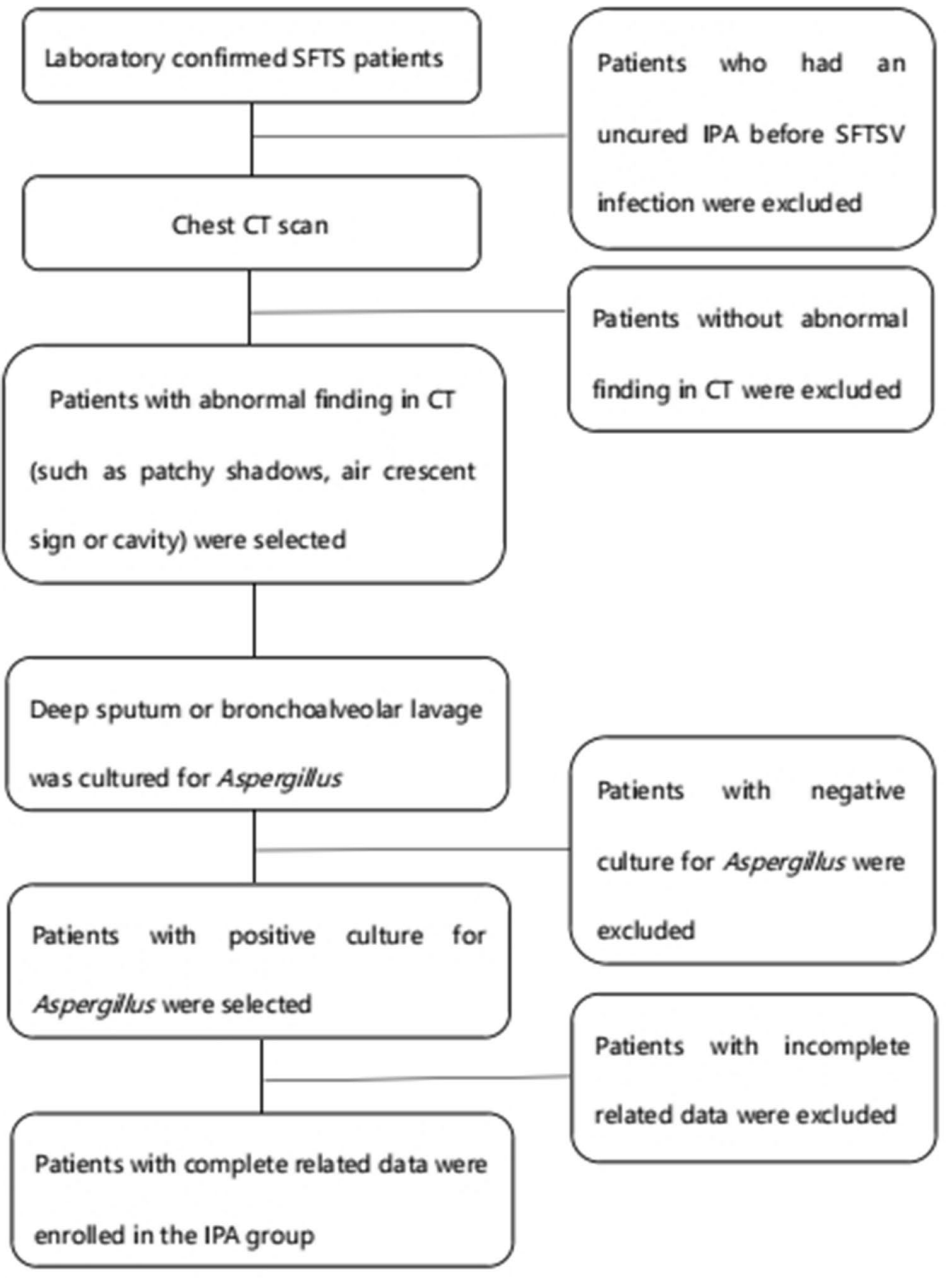
Flow chart of patients’ selection in IPA group.

The enrolled SFTS patients were further divided into IPA group (n = 22) and non-IPA group (n = 45). The formulation of diagnostic criteria for IPA was based on the 2019 European Organization for the Research and Treatment of Cancer/Mycosis Study Group (EORTC/MSG) consensus^11^: (i) compatible signs and symptoms of IPA (such as cough, expectoration or wheezing); (ii) abnormal findings by CT scan of the lungs (such as patchy shadows, air crescent sign or cavity formation); (iii) mycological evidence: positive culture for *Aspergillus* from deep sputum or bronchoalveolar lavage. Patients who met all three or the last two criteria were diagnosed as IPA. The exclusion criteria included: (i) the patients had an uncured IPA before SFTSV infection; (ii) the related data of patients were incomplete because of death or other reasons. Patients who met any of the exclusion criteria were excluded. The flow chart of patients' selection was shown in Fig. 1.

### Data collection

Related data of enrolled SFTS patients including demographic feature, clinical manifestations and laboratory parameters were collected and sorted according to their electronic medical records. Among clinical manifestations, uncontrolled diabetes and central nervous system (CNS) symptoms were defined as follows. Uncontrolled diabetes is defined as the condition that the fasting blood glucose is still higher than 7.0 mmol/L despite the treatment with oral medicine or subcutaneous injection of insulin. CNS symptoms are defined as the presence of restlessness, lethargy or coma. All authors had access to information that could identify individual participants during or after data collection.

### Statistical analysis

Statistical analysis was conducted using SPSS software (version 26.0). Categorical variables were represented by rate. The measurement data of normal and abnormal distribution were compared by t-test and Wilcoxon rank sum test, respectively. Enumeration data were compared by chi-square test or Fisher exact probability test. Univariate analysis was performed to assess the relevance between demographic feature, clinical manifestations, laboratory parameters and occurrence of IPA. Factors with P < 0.05 in univariate analysis were further analyzed using multivariable logistic regression analysis to identify the independent risk factors for IPA. Receiver operating characteristic (ROC) curve analysis was used to evaluate the sensitivity and specificity of independent risk factors to predict IPA. For all analysis, a P-value less than 0.05 was considered statistically significant.

### Ethics approval and consent to participate

This study was approved by Medical Ethical Committee in Qilu Hospital of Shandong University (2021–120). Written informed consent was acquired from every enrolled patients or their guardians.

## Results

### Demographic characteristics

During the period of this study from April 2021 to August 2022, a total of 67 laboratory confirmed SFTS patients were enrolled. Average age of the patients was 64.7 (± 8.4) years old, and 34 (50.7%) were male. Among the 67 patients, 22 (32.8%) were diagnosed with IPA, based on which they were assigned to the IPA group. The average time from onset of SFTS illness to IPA diagnosis was 9.2 (± 2.3) days. The average age of the patients was 66.1 (± 7.2) years old, including 11 (50.0%) male patients and 11 (50.0%) female patient. 45 (67.2%) patients with an average age of 64.0 (± 8.9) years old were in non-IPA group, including 23 (51.1%) males and 22 (48.9%) females. The differences of age and gender between the two groups were not statistically significant (P = 0.341 and P = 0.932, respectively).

### Clinical manifestations

As shown in Table 1, mortality of SFTS patients in IPA group and non-IPA group was 27.3% (6/22) and 8.9% (4/45), respectively. The difference of mortality between the two groups was statistically significant (P = 0.047). Diabetes was the most relevant underlying disease to invasive aspergillosis. The incidences of diabetes among SFTS patients in IPA group and non-IPA group were 40.9% (9/22) and 31.1% (14/45), respectively. Rate of uncontrolled diabetes in IPA group was significantly higher than that in non-IPA group (31.8% vs 0.4%, P = 0.002). The average body temperature was 38.8 (± 0.6) °C in IPA group and 38.9 (± 0.6) °C in non-IPA group (shown in Table 1). The different degree of body temperature was not statistically significant (P = 0.493). In this study, the incidence of CNS symptoms in IPA group and non-IPA group was 72.7% (16/22) and 20.0% (9/45), respectively. A statistically significant difference between the two groups was observed (P < 0.001).

**Table 1:** Comparison of characteristics of SFTS patients between IPA group and Non-IPA group.

Characteristics	All patients (N = 67)	IPA group (N = 22)	Non-IPA group (N = 45)	P^a^ value
Age (mean ± SD, years)	64.7 ± 8.4	66.1 ± 7.2	64.0 ± 8.9	0.341
Gender				
Male	34 (50.7%)	11 (50.0%)	23 (51.1%)	0.932
Female	33 (49.3%)	11 (50.0%)	22 (48.9%)	
Mortality	10 (14.9%)	6 (27.3%)	4 (8.9%)	0.047
Underlying diseases				
Diabetes	23 (34.3%)	9 (40.9%)	14 (31.1%)	
Uncontrolled diabetes	9 (13.4%)	7 (31.8%)	2 (0.4%)	0.002
Clinical symptom				
Fever (°C)	38.9 ± 0.6	38.8 ± 0.6	38.9 ± 0.6	0.493
CNS symptoms	25 (37.3%)	16 (72.7%)	9 (20%)	< 0.001
Laboratory parameters				
WBC counts (× 10^9^/L)	3.1 ± 1.9	3.4 ± 2.7	2.9 ± 1.4	0.463
WBC < 2.0 × 10^9^/L	22 (32.8%)	9 (40.9%)	13 (28.9%)	0.325
Neutrophils counts (× 10^9^/L)	1.6 ± 1.1	1.6 ± 1.3	1.6 ± 1.0	0.999
Neutrophils < 1.5 × 10^9^/L	41 (61.2%)	14 (63.6%)	27 (45.0%)	0.774
Platelets counts (× 10^9^/L)	62.4 ± 53.0	33.9 ± 17.6	76.4 ± 58.8	0.002
Platelets < 40 × 10^9^/L	24 (35.8%)	15 (68.2%)	9 (20%)	< 0.001
CD4^+^ T cell counts/μL	348 ± 187	196 ± 107	423 ± 173	< 0.001
CD4^+^ T cell < 300/μL	30 (44.8%)	18 (81.8%)	12 (26.7%)	< 0.001
CD8^+^ T cell counts/μL	487 ± 308	287 ± 263	585 ± 283	< 0.001
CD8^+^ T cell < 400/μL	29 (43.3%)	18 (81.8%)	11 (24.4%)	< 0.001

^a^P value was obtained by comparing the data between IPA group and non-IPA group.

### Laboratory parameters

Some associated laboratory parameters including WBC, neutrophils, platelets, CD4^+^ T cell and CD8^+^ T cell were collected during the first seven days after onset of illness and the most severe values were selected to analyze. The results were shown in Table 1.

The counts of WBC, neutrophils and platelets were reduced obviously. The average counts of WBC and neutrophils in IPA group were 3.4 (± 2.7) × 10^9^/L and 1.6 (± 1.3) × 10^9^/L, respectively. The average counts of WBC and neutrophils in non-IPA group were 2.9 (± 1.4) × 10^9^/L and 1.6 (± 1.0) × 10^9^/L, respectively. The differences of counts of WBC and neutrophils between the two groups were not statistically significant (P = 0.463 and P = 0.999). The average counts of platelets in IPA group was statistically lower than that in non-IPA group (33.9 (± 17.6) × 10^9^/L vs 76.4 (± 58.8) × 10^9^/L, P = 0.002).

The counts of CD4^+^ and CD8^+^ T cell in IPA group were 196 (± 107)/μL and 287 (± 263)/μL, while the counts of CD4^+^ and CD8^+^ T cell in non-IPA group were 423 (± 173)/μL and 585 (± 283)/μL. The differences of counts of CD4^+^ and CD8^+^ T cell between IPA group and non-IPA group were statistically significant (P < 0.001). The incidence of CD4^+^ T cell < 300/μL and CD8^+^ T cell < 400/μL in IPA group was 81.8% (18/22), while the incidences of CD4^+^ T cell < 300/μL and CD8^+^ T cell < 400/μL in non-IPA group were 26.7% (12/45) and 24.4% (11/45). The differences of incidences of CD4^+^ T cell < 300/μL and CD8^+^ T cell < 400/μL between IPA group and non-IPA group were statistically significant (P < 0.001).

### Multivariable logistic regression analysis

Five variables were found to be statistically different (P < 0.05) based on the univariate risk assessment. To identify the independence of each variable for promotion of IPA, multivariable logistic regression analysis were performed. As shown in Table 2, the results of multivariable logistic regression analysis indicated uncontrolled diabetes, platelets < 40 × 10^9^/L, CD4^+^ T cell < 300/μL, and CD8^+^ T cell < 400/μL were associated with occurrence of IPA in SFTS patients.

**Table 2:** Multivariable logistic regression analysis on statistically significant variables.

Variables	B^a^	SE^b^	Wald^c^	P value	OR	95% CI
Uncontrolled diabetes	1.387	0.656	4.464	0.035	4.003	1.106–14.491
CNS symptoms	0.318	0.924	0.118	0.731	1.374	0.224–8.409
Platelets < 40 × 10^9^/L	2.344	0.899	6.796	0.009	10.424	1.789–60.733
CD4^+^ T cell < 300/μL	1.753	0.846	4.290	0.038	5.772	1.099–30.319
CD8^+^ T cell < 400/μL	2.399	0.995	5.812	0.016	11.013	1.566–77.439

^a^Regression coefficient; ^b^Standard error; ^c^Chi-square value.

### Receiver operating characteristic curve analysis

**Figure 2:**
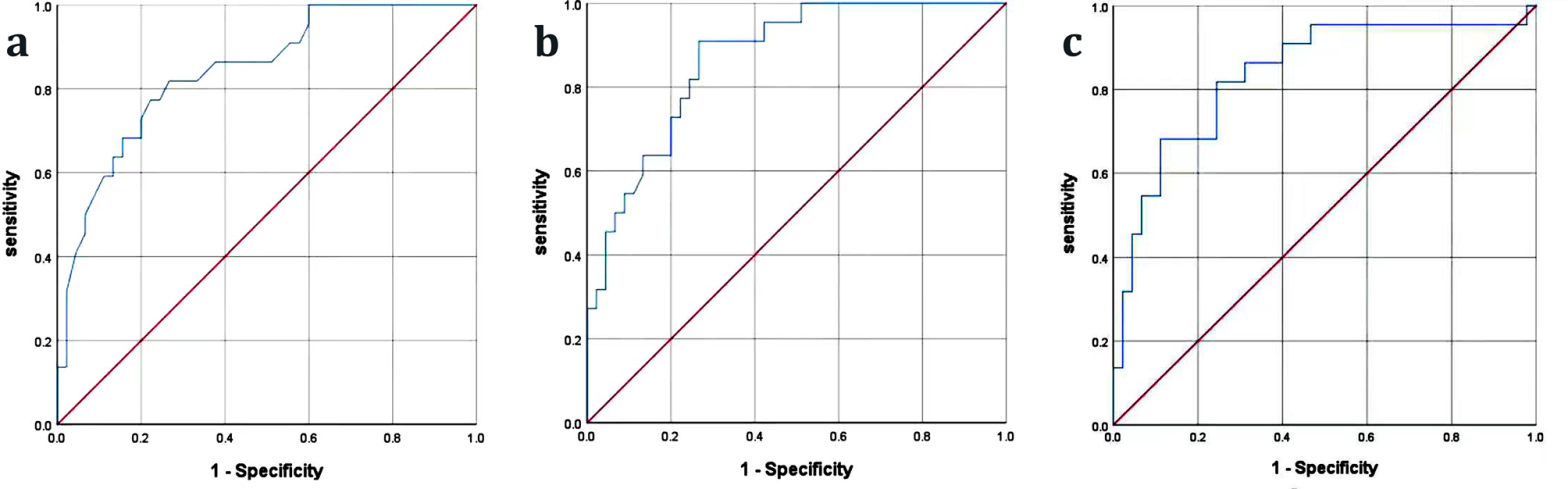
Receiver operating characteristic (ROC) curve analysis of predictors for IPA in patients with SFTS. (**a**) ROC curve of platelets (cutoff value: 45; area under curve: 0.841; 95% CI 0.743–0.940; sensitivity: 81.8%; specificity: 73.3%; P < 0.001).; (**b**) ROC curve of CD4^+^ T cell (cutoff value: 319; area under curve: 0.868; 95% CI 0.784–0.952; sensitivity: 90.9%; specificity: 73.3%; P < 0.001); (**c**) ROC curve of CD8^+^ T cell (cutoff value: 395; area under curve: 0.837; 95% CI 0.729–0.946; sensitivity: 81.8%; specificity: 75.6%; P < 0.001).

As showed in Table 3 and Fig. 2, ROC curve analysis was used to evaluate the sensitivity and specificity of independent factors to predict IPA in SFTS patients. Cut-off value of platelets to predict IPA incidence in SFTS patients was 45 × 10^9^/L, with sensitivity of 81.8% and specificity of 73.3%. Cut-off value of CD4^+^ T cell counts to predict IPA was 319 cells/μL, with sensitivity of 90.9% and specificity of 73.3%. Cut-off value of CD8^+^ T cell counts to predict IPA was 395 cells/μL, with sensitivity of 81.8% and specificity of 75.6%.

**Table 3:** Receiver operating characteristic curve analysis of independent predictors for IPA in SFTS patients.

Parameters	Cutoff value	Area under curve (95% CI)	Sensitivity (%)	Specificity (%)	P value
Platelets (10^9^/L)	45	0.841 (0.743–0.940)	81.8	73.3	< 0.001
CD4^+^ T cell (cells/μL)	319	0.868 (0.784–0.952)	90.9	73.3	< 0.001
CD8^+^ T cell (cells/μL)	395	0.837 (0.729–0.946)	81.8	75.6	< 0.001

## Discussion

Our previous study on the risk factors associated with fatal outcome showed that pulmonary infection was significantly associated with risk of death among SFTS patients^12^. In this study, mortality of SFTS patients in IPA group was significantly higher than that in non-IPA group. The result indicated that IPA could be considered a risk factor of fatal outcome and early identification of IPA among SFTS patients was necessary. In this study, the incidence of IPA in SFTS patients was 32.8%, which was similar to the studies from Bae et al. and Xu et al. with incidence of 20% and 31.9%, respectively^8,10^. These results indicated SFTS patients would be more prone to aspergillus infection. Some factors might contribute to increased risks of susceptibility to IPA, including median age ˃ 60 years, uncontrolled underlying disease, leukocytopenia and neutropenia, severe complications, impaired immune functions and excessive inflammatory response. The aim of this study was to confirm predictors for IPA in their early course of the disease by analyzing demographic feature, clinical manifestations and laboratory parameters of SFTS patients.

Patients with diabetes are prone to aspergillus infection because high level of blood glucose is conducive to the growth of aspergillus, inhibits leukocyte chemotaxis, reduces phagocytosis of phagocytes, and decreases complement production^13^. Diabetes may be considered a risk factor for the development of aspergillus infection and should be added to the list of well-known risk factors for invasive aspergillosis^14^. In our study, diabetes was a common underlying disease in SFTS patients with a rate of 34.3%. Although the proportion of patients with diabetes in IPA group was similar to that in non-IPA group, uncontrolled diabetes were more common in IPA group with a rate of 31.8%. The difference of incidence of uncontrolled diabetes between the two groups was statistically significant, which suggested that uncontrolled diabetes might be a risk factor for the development of IPA. The result of multivariable logistic regression analysis further confirmed that uncontrolled diabetes was an independent predictor for IPA. The conclusion was not consistent to the study by Xu et al.^15^, in which diabetes was recognized as a risk factor for IPA with univariate analysis and not an independent risk factor by multivariable logistic regression analysis. The reason might be that the previous study regarded diabetes as a risk factor to analyze, but did not analyze the control condition of diabetes.

CNS symptoms are one of the most common severe complications among SFTS patients and has been confirmed to be associated with fatal outcome^2,12^. In our study, the incidence of CNS symptoms was 37.3%. Sixteen patients in IPA group had CNS symptoms, which was significantly higher than that in non-IPA group. Patients with CNS symptoms might have intestinal flora disturbance and bucking, which would increase the risk for pulmonary infection^16^. The significant difference of incidence of CNS symptoms between the two groups indicated that combined with CNS symptoms could be considered a risk factor for IPA. However, multivariable logistic regression analysis did not support it as an independent predictor for IPA.

Leukopenia, mainly neutropenia, is a well-known risk factor for IPA. For invasive aspergillus infection, neutrophils are the major immune cells in non-specific immunity and exert anti-infection effects through chemotaxis, opsonization and phagocytosis. Therefore, persistent neutropenia is a high risk for deep aspergillus infection. Platelets play anti-fungal effect by adhering to the cell wall of the hyphae to block aspergillus germination and hyphal elongation^17^. When activated platelets was removed from blood, thrombocytopenia may result in invasive aspergillus infection^18^. SFTS patients are characterized by leukopenia and thrombocytopenia, which may be high risk factors for IPA. In the present study, the counts of WBC and neutrophils were reduced obviously in both IPA group and non-IPA group, and there was no significant difference between the two groups. Therefore, the results did not suggest that reduced WBC and neutrophils were risk factors for IPA among SFTS patients. The reason might be that leukopenia and neutropenia were transient which were not like patients with hematologic tumor^19^. Although thrombocytopenia could be seen in both groups, thrombocytopenia was more pronounced in IPA group than that in non-IPA group and the difference was significant. Furthermore, the proportion of patients with platelets < 40 × 10^9^/L in IPA group was higher than that in non-IPA group. These results indicated that thrombocytopenia might be a predictor for IPA, which was confirmed by multivariable logistic regression analysis. Especially, when the cutoff value of platelets was 45 × 10^9^/L, the sensitivity and specificity for predicting IPA were 81.8% and 73.3%.

Most SFTS patients are elderly with the median age of 63 years and immune function decreases with the increase of age, which is associated with the severity and mortality of the disease^20^. In addition, SFTSV infection can also damage the immune function of SFTS patients through changing the distribution of lymphocytic sub-populations^7^. These reasons put patients at high risk for aspergillus infection. In this study, age was not an independent predictor for IPA, but the damage of immune function due to SFTSV infection could be considered an independent predictor. Generally, cell mediated immunity plays powerful roles in protection against invasive fungal infection. CD4^+^ T cell, as antigen presenting cell and CD8^+^ T cell, as a cytotoxic cell constitute an important immune defense barrier against fungal infection. In this study, CD4^+^ and CD8^+^ T cells decreased obviously in IPA group with an average level of 196(± 107)/μL and 287 (± 263)/μL, respectively. The decreased T lymphocytes resulted in the number of active T cells were insufficient to participate in cellular immune response and caused lower cellular immune function among SFTS patients, which increased the risk of IPA. The result of multivariable logistic regression analysis suggested that CD4^+^ T cell < 300/μL and counts of CD8^+^ T cell < 400/μL could be considered as independent predictors for development of IPA. The results were consistent to the previous study^21^, in which decreased lymphocyte was also considered a predictive factor for IPA among SFTS patients. Base on the ROC curve analysis, the sensitivity and specificity for predicting IPA were higher when the counts of CD4^+^ T cell < 319/μL and counts of CD8^+^ T cell < 395/μL.

However, the present study had some limitations. First, based on EORTC/MSG criteria, the diagnosis of IPA in this study was categorized into probable diagnosis for lack of histopathologic evidence. False positive might be present among patients diagnosed with IPA. In the previous study^22^, a clinical algorithm for diagnosing IPA by discriminating *Aspergillus* colonization from invasive disease in ICU patients with *Aspergillus* positive cultures was established, and the algorithm demonstrated 61% specificity and 92% sensitivity. Similar to the previous study^22^, better diagnosis methods are necessary to discriminate colonization from FSTS patients who were positive for *Aspergillus* culture, and to increase the rigor of the research. Second, corticosteroids had been clinically applied to treat SFTS patients because of the ability to suppress systemic inflammation response and alleviate cytokine storm. However, inappropriate application of corticosteroids for treatment in SFTS patients may cause secondary infection^23^, which contributes to the development of IPA. Use of corticosteroids was not included in analysis due to the low rate, low dosage and short duration (< 3 days) of use among the enrolled SFTS patients. Third, rapid replication of virus may result in imbalance of immune regulation, which makes SFTS patients susceptible to IPA. Comparison of viral load between IPA group and non-IPA group was not analyzed because the examination of viral load in most patients was missing. Further investigations are needed on the limitations to confirm whether these factors are risk predictors for development of IPA among patients with SFTS.

## Conclusion

Patients with SFTS need to be alert to the possibility of secondary IPA. Our study confirmed that uncontrolled diabetes, platelets < 45 × 10^9^/L, CD4^+^ T cell < 319/μL and CD8^+^ T cell < 395/μL could be considered independent predictors for development of IPA. Identification of these predictors may prompt physicians to initiate early diagnostic examinations for aspergillus infection and to initiate initial treatment, which contribute to improve outcomes of SFTS patients.

## Data Availability

The databases used and analyzed during the current study are available from the corresponding author on reasonable request.

## Abbreviations

SFTS: Severe fever with thrombocytopenia syndrome
SFTSV: Severe fever with thrombocytopenia syndrome virus
IPA: Invasive pulmonary aspergillosis
ROC: Receiver operating characteristic
RT-PCR: Real-time polymerase chain reaction
CNS: Central nervous system

## References

[1] Yu XJ, Liang MF, Zhang SY, Liu Y, Li JD, Sun YL (2011). Fever with thrombocytopenia associated with a novel bunyavirus in China. N. Engl. J. Med..

[2] Gai ZT, Zhang Y, Liang MF, Jin C, Zhang S, Zhu CB (2012). Clinical progress and risk factors for death in severe fever with thrombocytopenia syndrome patients. J. Infect. Dis..

[3] Liu K, Zhou H, Sun RX, Yao HW, Li Y, Wang LP (2015). A national assessment of the epidemiology of severe fever with thrombocytopenia syndrome, China. Sci. Rep..

[4] Huang X, Li J, Li A, Wang S, Li D (2021). Epidemiological characteristics of severe fever with thrombocytopenia syndrome from 2010 to 2019 in Mainland China. Int. J. Environ. Res. Public Health..

[5] Liu JW, Fu HM, Sun DP, Wu SZ, Wang L, Yao MX (2020). Analysis of the laboratory indexes and risk factors in 189 cases of severe fever with thrombocytopenia syndrome. Medicine (Baltimore).

[6] Bassetti M, Garnacho-Montero J, Calandra T, Kullberg B, Dimopoulos G, Azoulay E (2017). Intensive care medicine research agenda on invasive fungal infection in critically ill patients. Intensive Care Med..

[7] Sun LP, Hu YJ, Niyonsaba A, Tong QX, Lu L, Li HY (2014). Detection and evaluation of immunofunction of patients with severe fever with thrombocytopenia syndrome. Clin. Exp. Med..

[8] Bae S, Hwang HJ, Kim MY, Kim MJ, Chong YP, Lee SO (2020). Invasive pulmonary aspergillosis in patients with severe fever with thrombocytopenia syndrome. Clin. Infect. Dis..

[9] Chen X, Yu Z, Qian Y, Dong DJ, Hao YY, Liu N (2018). Clinical features of fatal severe fever with thrombocytopenia syndrome that is complicated by invasive pulmonary aspergillosis. J. Infect. Chemother..

[10] Xu Y, Shao MR, Liu N, Tang J, Gu Q, Dong DJ (2021). Invasive pulmonary aspergillosis is a frequent complication in patients with severe fever with thrombocytopenia syndrome: A retrospective study. Int. J. Infect. Dis..

[11] Donnelly JP, Chen SC, Kauffman CA, Steinbach WJ, Baddley JW, Verweij PE (2020). Revision and update of the consensus definitions of invasive fungal disease from the European Organization for Research and Treatment of Cancer and the Mycoses Study Group Education and Research Consortium. Clin. Infect. Dis..

[12] Song L, Zhao YJ, Wang G, Huang DY, Sai LT (2022). Analysis of risk factors associated with fatal outcome among severe fever with thrombocytopenia syndrome patients from 2015 to 2019 in Shandong, China. Eur. J. Clin. Microbiol..

[13] Trof RJ, Beishuizen A, Debets-Ossenkopp YJ, Girbes ARJ, Groeneveld ABJ (2007). Management of invasive pulmonary aspergillosis in non-neutropenic critically ill patients. Intensive Care Med..

[14] Farhad G, John AT (2017). Weight loss and diabetes are new risk factors for the development of invasive aspergillosis infection in non-immunocompromized humans. Clin. Pract. (Lond)..

[15] Xu Y, Shao MR, Liu N, Tang J, Gu Q, Dong DJ (2021). Invasive pulmonary aspergillosis is a frequent complication in patients with severe fever with thrombocytopenia syndrome: A retrospective study. Int. J. Infect. Dis..

[16] Zhao J, Li LQ, Zhen NX, Du LL, Shan H, Yu Y (2021). Microbiology and outcomes of institutionalized patients with stroke-associated pneumonia: An observational cohort study. Front. Microbiol..

[17] Speth C, Hagleitner M, Ott HW, Wurzner R, Lass-Florl C, Rambach G (2013). Aspergillus fumigatus activates thrombocytes by secretion of soluble compounds. J. Infect. Dis..

[18] Deshmukh H, Speth C, Sheppard DC, Neurauter M, Wurzner R, Lass-Florl C (2020). Aspergillus-derived galactosaminogalactan triggers complement activation on human platelets. Front. Immunol..

[19] Livio P, Alessandro B, Anna C, Chiara C, Simone C, Rosa F (2017). Risk stratification for invasive fungal infections in patients with hematological malignancies: SEIFEM recommendations. Blood Rev..

[20] Li JC, Zhao J, Li H, Fang LQ, Liu W (2022). Epidemiology, clinical characteristics, and treatment of severe fever with thrombocytopenia syndrome. Infect. Med..

[21] Hu LF, Kong QX, Yue CC, Xu XH, Xia LL, Bian TT (2021). Early-warning immune predictors for invasive pulmonary aspergillosis in severe patients with severe fever with thrombocytopenia syndrome. Front. Immunol..

[22] Stijn IB, Fabio ST, Van den Abeele A-M (2012). A clinical algorithm to diagnose invasive pulmonary aspergillosis in critically ill patients. Am. J. Respir. Crit. Care Med..

[23] Seo JW, Kim D, Yun N, Kim DM (2021). Clinical update of severe fever with thrombocytopenia syndrome. Viruses.

